# A Novel Approach to Addressing an Unintended Consequence of Direct to Room: The Delay of Initial Vital Signs

**DOI:** 10.5811/westjem.2017.12.35068

**Published:** 2018-02-19

**Authors:** Joseph Basile, Elias Youssef, Bartholomew Cambria, Jerel Chacko, Karyn Treval, Barry Hahn, Brahim Ardolic

**Affiliations:** Northwell Health, Staten Island University Hospital, Department of Emergency Medicine, Staten Island, New York

## Abstract

**Introduction:**

The concept of “direct to room” (DTR) and “immediate bedding” has been described in the literature as a mechanism to improve front-end, emergency department (ED) processing. The process allows for an expedited clinician-patient encounter. An unintended consequence of DTR was a time delay in obtaining the initial set of vital signs upon patient arrival.

**Methods:**

This retrospective cohort study was conducted at a single, academic, tertiary-care facility with an annual census of 94,000 patient visits. Inclusion criteria were all patients who entered the ED from 11/1/15 to 5/1/16 and between the hours of 7 am to 11 pm. During the implementation period, a vital signs station was created and a personal care assistant was assigned to the waiting area with the designated job of obtaining vital signs on all patients upon arrival to the ED and prior to leaving the waiting area. Time to first vital sign documented (TTVS) was defined as the time from quick registration to first vital sign documented.

**Results:**

The pre-implementation period, mean TTVS was 15.3 minutes (N= 37,900). The post-implementation period, mean TTVS was 9.8 minutes (N= 39,392). The implementation yielded a 35% decrease and an absolute reduction in the average TTVS of 5.5 minutes (*p*<0.0001).

**Conclusion:**

This study demonstrated that the coupling of registration and a vital signs station was successful at overcoming delays in obtaining the time to initial vital signs.

## INTRODUCTION

The concept of “direct to room” (DTR), also known as “immediate bedding,” has been reported in the literature as a mechanism to improve front-end emergency department (ED) processing.^1^ At one institution DTR was referred to as “closing” the waiting room, since patients were taken directly to a bed, when available, without undergoing formal triage and registration in the waiting room.^2^ Reducing wait times has been linked to patient perceptions of superior service and increased trust, especially in private hospitals.^3^ Although every ED may have individual front-end processes, most ED visits include patient presentation, registration, triage, bed assignment, and medical evaluation.^4^

Various models have been implemented in an attempt to reduce ED wait times and overall length of stay (LOS), from split flows to rapid triage.^5^,^6^,^7^ DTR uses the design of parallel processing, as opposed to serial processing, which allows patients to bypass many preliminary steps between arrival to the ED and placement in a bed. The goal is to decrease the backlog of waiting room patients waiting for less-critical tasks and allow registration, nursing evaluation, and medical provider evaluation to occur simultaneously at the bedside.^4^,^8^ More importantly, this facilitates an expedited clinician and patient interaction.

The literature suggests that DTR can decrease waiting times, ED LOS, and left without being seen (LWBS) rates, while simultaneously improving patient satisfaction.^4^ Bertoty et. al. reported that the LOS for admitted patients decreased by 7.7%, and the LOS for discharged patients also decreased after DTR was implemented.^1^ Similarly, there was an improvement in patient satisfaction, which was hypothesized to occur since patients prefer to wait in a treatment area rather than a waiting room. Patients also perceived their treatment as beginning from the moment they were brought into the treatment area.

At our institution, we implemented a DTR policy, which improved our front-end process dramatically. The Staten Island University Hospital ED has seen an improvement in metrics, similar to those cited in the literature, since implementing a DTR process. This includes decreased physician turn-around time, a decrease in LWBS, and a marked increase in patient satisfaction. Unfortunately, such improvements were accompanied by unforeseen consequences. In a traditional system, all patients undergo a formal triage process by a dedicated nurse, during which vital signs are obtained. In the DTR process, this step may be bypassed. Consequently, we noticed a delay from the time of presentation to the first recorded set of vital signs. In some circumstances, patients were unwittingly treated and released before obtaining a single set of vital signs. To address this issue, we developed a vital signs station within the waiting area. Our goal was to determine the feasibility and effectiveness of obtaining and recording vital signs within 10 minutes of every patient’s arrival to the ED after initiation of a DTR process.

## METHODS

This retrospective, cohort study took place at a single, academic, tertiary-care, Level I trauma center with an annual census of approximately 94,000 visits. Inclusion criteria were all patients who entered the ED between the hours of 7 am to 11pm. We excluded from the study all patients who entered the ED between 11 pm and 7 am. due to inability to staff the vital signs station during these hours of the pilot phase of the program. The pre-implementation time period used for comparison was November 1, 2014, to May 1, 2015. The post-implementation time period was November 1, 2015, to May 1, 2016. We defined TTVS documented as the time from quick registration to first vital sign documented in the electronic medical record (EMR). The pilot phase was initiated in May 2014 for eight hours/day, five days/week, excluding weekends. This was extended to 16 hours/day, seven days/week in November 2014, which was the study period.

**Population Health Research Capsule**

What do we already know about this issue?

The direct-to-room (DTR) concept uses parallel processing to decrease ED wait times, length of stay, and left without being seen rates, but may result in vital sign delays.

What was the research question?

Does a vital signs station in the waiting room reduce the time to first vital signs to under 10 minutes?

What was the major finding of the study?

A vital signs station in the waiting room reduced the mean time to first vital signs by 5.5 minutes, a 35% reduction.

How does this improve population health?

This improves front-end ED processing by maintaining all of the advantages of DTR without delaying initial vital signs, which improves patient safety.

During the implementation period, a vital signs station was created and a personal care assistant (PCA) was assigned to the waiting area with the designated job of obtaining vital signs on all patients upon arrival to the ED and prior to leaving the waiting area. PCAs are part of the ED team and perform duties under the supervision of doctors and nurses. They assist with numerous tasks. This vital sign station was directly adjacent to the quick registration desk. After patient arrival and sign-in, a quick registration including name, date of birth, and chief complaint was completed. Subsequently, patients were directed to a PCA with a portable vital signs machine and a computer on wheels with access to the EMR. The PCA’s sole task was to obtain vital signs on all patients before they left the waiting area and then enter this information in the EMR. Patients who arrived via EMS had vital signs entered by the ED triage nurse and were also included in this analysis. PCAs were also empowered to obtain vital signs on patients who were waiting in line for registration.

## STATISTICAL ANALYSIS

We reported summary statistics as mean ± standard deviation and median (first quartile, third quartile) for the continuous variable TTVS. We compared the difference between pre-implementation and post-implementation periods in the primary outcome variable of TTVS with the Wilcoxon two-sample test. All statistical tests are two-sided, and a p-value of <0.05 was considered to indicate statistical significance. We performed all statistical analyses using the SAS software, Version 9.3 (SAS Inc., Cary, NC, USA).

## RESULTS

The total census between November 1, 2014, and May 1, 2015, was 44,177 patients. The total census between November 1, 2015, and May 1, 2016, was 45,807 patients. During the study period, 37,900 subjects were enrolled in the control group (pre-implementation group) and 39,392 subjects were enrolled in the intervention group (post-implementation group). The pre-implementation period mean TTVS was 15.3 minutes (N= 37,900) with a median of 9.0 minutes and a range of 0 to 846 minutes. The post-implementation period mean TTVS was 9.8 minutes (N= 39,392) with a median of 5.0 minutes and a range of 0 to 479 minutes. The implementation yielded an average TVVS reduction of 5.5 minutes (*p*<0.0001), a 35% reduction.

## DISCUSSION

The implementation of DTR has had countless benefits, including faster turnaround times, improved door-to-doctor times, and decreased LWBS rates.^3^ By reducing ED crowding, decision-making time can be reduced as well as reducing over-use of the laboratory and computed tomography.^9^ However, our experience has shown that an unintended consequence of DTR is both a delay and inconsistency in obtaining initial vital signs. In this study, we demonstrated that the implementation of a vital sign station at ambulatory registration reduced the TTVS, an unintended consequence of DTR, by a mean time of nearly six minutes.

When we coupled a vital signs station with our already-existing quick registration process, the department experienced no delays in overall throughput. Although this now adds a few minutes to the quick registration, we found that the overall benefits far outweigh this short delay. For EDs that have some form of quick registration and DTR process and experience similar delays in obtaining vital signs, we believe that creating a vital sign station in the waiting room is a feasible and effective solution that could be implemented by any ED.

Our ED has two portals of entry: an ambulance entrance, where the patient is immediately triaged and has his vital signs obtained by a nurse who then enters them in the patient chart; and a quick registration desk in the waiting room where all ambulatory patients must sign in prior to being brought to the treatment area. At the quick registration desk, brief demographic information and chief complaint is obtained, which allows the patient to be entered into the EMR and receive a medical record number. After undergoing a quick registration, there are three subsequent pathways for the patient: 1) taken directly into the treatment area by a nurse, PCA, or pavilion coordinator (our DTR process); 2) taken to a triage station for formal nursing triage, 3) queued in the waiting room for either the next available DTR or formal triage availability.

At our institution the pavilion coordinator is an ED greeter who helps the nursing staff facilitate our DTR process. Quick registration with chief complaint and vital sign assessment is markedly different from formal triage, in that formal triage requires nursing resources and a significant amount of time. Quick registration only requires patient demographics and chief complaint, whereas traditional formal triage includes expanded history-taking and a medical assessment including allergies, medications, surgical history, etc. which can lead to a delay in initial clinical assessment in treatment areas.

There are many potential benefits to this new process besides the decrease in TTVS. Obtaining earlier vital signs enhances patient safety since it allows for earlier recognition of potentially abnormal vital signs and therefore prompt treatment and intervention. This is especially true in the patient who may appear stable. Second, patient satisfaction is improved since they recognize that they are being taken care of from the moment they walk into the ED. Implementation may be limited due to PCA competing priorities and unanticipated staffing needs within the department. While there were no extra personnel costs as staffing did not increase to fill the vital signs station, we did decrease the availability of existing PCAs in the clinical arena.

## LIMITATIONS

This study has several limitations. Because it was performed at a single ED, the results may not be duplicated or applicable at another ED. In addition, the study was retrospective, and therefore results are subject to the biases associated with a retrospective study. Also, enrollment in the study was limited to 7 am – 11 pm due to limitations in staffing outside of this time frame.

We included in the analysis patients who arrived via EMS during the study period. The electronic report generated for this project does not have a mechanism to separate EMS from non-EMS patient arrivals. This report identifies all ED patients and generates a time from arrival to first vital sign. Our EMS process did not change in the study periods and we have no reason to believe that this would have had any impact on our results. Of note, between 2014–2016 our annual EMS arrivals have been consistently 20% of our overall volume. Given that our study period included a seasonal comparative as a control and there were no changes in the departmental management of EMS triage, we do not believe that this would have had an effect on our results.

Outliers were noted in both groups. We can only hypothesize that these delays were likely secondary to poor provider documentation. The report generated notes the first time vital signs were documented in the EMR. This is not an absolute reflection of what may have taken place. For example, if vital signs were obtained earlier on in a visit and noted by a provider but inadvertently were not placed into the chart in a timely manner, it’s easy to see how any outlier could occur.

## CONCLUSION

This study found that coupling quick registration to a vital signs station in the waiting room is both a feasible and effective method to overcome delays in obtaining initial vital signs in a “direct-to-room” ED process.

## Figures and Tables

**Figure:**
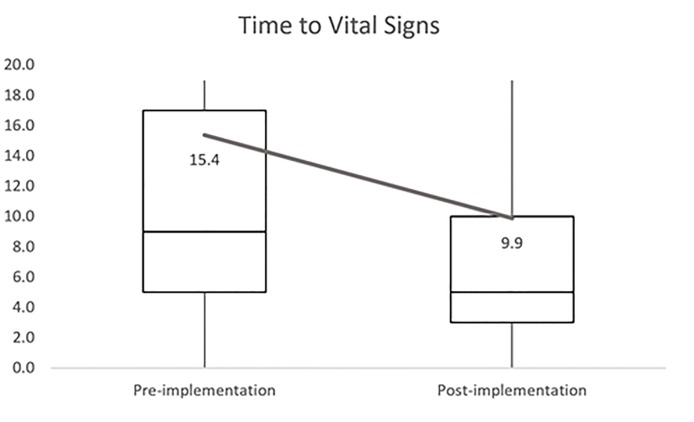
Time (minutes) to vital signs first recorded demonstrated as box-and-whisker plot, modified with maximum values shown at tops of curtailed whiskers. Mean values are demonstrated with trendline.

**Table:** Characteristics of patients whose initial vital signs were obtained in the waiting room as part of an existing quick-registration process.

	Pre-intervention	Post-intervention
Age	41.9 (25.3)	41.6 (25.1)
Males (%)	47.4%	47.1%
ESI		
1	0.2%	0.6%
2	1.4%	2.9%
3	40.9%	47.4%
4	52.6%	46.1%
5	4.2%	2.4%
unassigned	0.8%	0.6%

ESI, Emergency Severity Index.
